# A Rare Presentation of Drug-induced Liver Injury with Fluticasone and Vilanterol Inhaler Use

**DOI:** 10.7759/cureus.4933

**Published:** 2019-06-18

**Authors:** Mohit Rishi, Amanda Wagner, Mark Ulanja, Bryce D Beutler, Karmjit Singh

**Affiliations:** 1 Internal Medicine, University of Nevada, Reno School of Medicine, Reno, USA; 2 Family Medicine, University of Nevada, Reno School of Medicine, Reno, USA; 3 Internal Medicine, Aureus University School of Medicine, Oranjestad, ABW

**Keywords:** drug induced liver injury, breo ellipta, idiosyncratic, dili

## Abstract

Drug-induced liver injury (DILI) is a rare and potentially lethal condition associated with the use of many commonly-used medications, including inhaled fluticasone-vilanterol. Therefore, a careful review of medications should always be obtained in the setting of acute onset hepatic dysfunction. We present the first reported case of idiosyncratic drug-induced liver injury associated with the use of this medication.

## Introduction

Drug-induced liver injury (DILI) represents hepatocellular damage after exposure to medication or supplements. Data from population-based studies suggest an approximate incidence of around 13.9 to 19.1 per 100,000 people [1]. The true incidence of DILI is difficult to discern because of an unknown population receiving offending drugs, difficulty assigning causation, and the absence of a standardized reporting system [2]. Drug hepatotoxicity can represent a predictable dose-dependent response to a given agent or occur as idiosyncratic DILI. While acetaminophen is the most commonly associated medication with a predictable injury response, idiosyncratic DILI is associated with medications such as amoxicillin/clavulanate, isoniazid, and nonsteroidal anti-inflammatory agents [3]. DILI is categorized as hepatocellular injury, cholestatic, or mixed presentation on the basis of liver biochemical markers. Risk factors for susceptibility injury include genetic, environmental (eg - alcohol consumption), and compound-related factors including lipophilia as a predominant characteristic of medications which trigger drug-induced liver injury [3]. Idiosyncratic DILI often goes unrecognized in the Food and Drug Administration (FDA) trials due to its rarity [4,5]. Furthermore, herbal and dietary supplements are not subject to FDA mandated scrutiny, testing, and monitoring in the post-marketing period. Dietary supplements that have been associated with DILI include flavocoxid, pyrrolizidine alkaloids, and prohormone supplements [3]. For the majority of the cases, full recovery is expected with removal of the inciting agent. However, hepatocellular injury patterns carry a worse prognosis or death in up to 10% of the cases, especially if associated with jaundice on presentation [6].

Here, we present a case of idiosyncratic DILI from the use of a fluticasone-vilanterol inhaler. This is a combination of inhaled corticosteroid and long-acting beta-2 adrenoreceptor agonist. This combination medication has been FDA-approved for chronic obstructive pulmonary disease. Commonly reported side effects include headache, diarrhea, hypertension, and oropharyngeal candidiasis. To our knowledge, there have been no other reports of DILI with the use of this combination medication.

## Case presentation

A 74-year-old Caucasian male presented to the emergency department with complaints of shortness of breath, nausea, diarrhea, generalized weakness, and jaundice. He reported that he had started fluticasone-vilanterol for the management of chronic obstructive pulmonary disease (COPD) three days prior to presentation. He denied a history of liver disease, alcohol use, over-the-counter supplement use, recent antibiotic use, herbal product use, or acetaminophen use. His past medical history included asthma, tobacco use disorder, and non-insulin-dependent type 2 diabetes mellitus. His outpatient medications included metformin, vitamin D supplement, low-dose aspirin, and a rescue albuterol inhaler. The patient was an ex-smoker with a 25 pack-year smoking history and quit tobacco nine months prior to presentation. He also denied any other illicit substance abuse such as marijuana, cocaine, and heroin. His family history was notable for COPD and chronic renal failure, but he denied liver disease in the family. He denied any recent travel or recent sick contacts.

**Figure 1:**
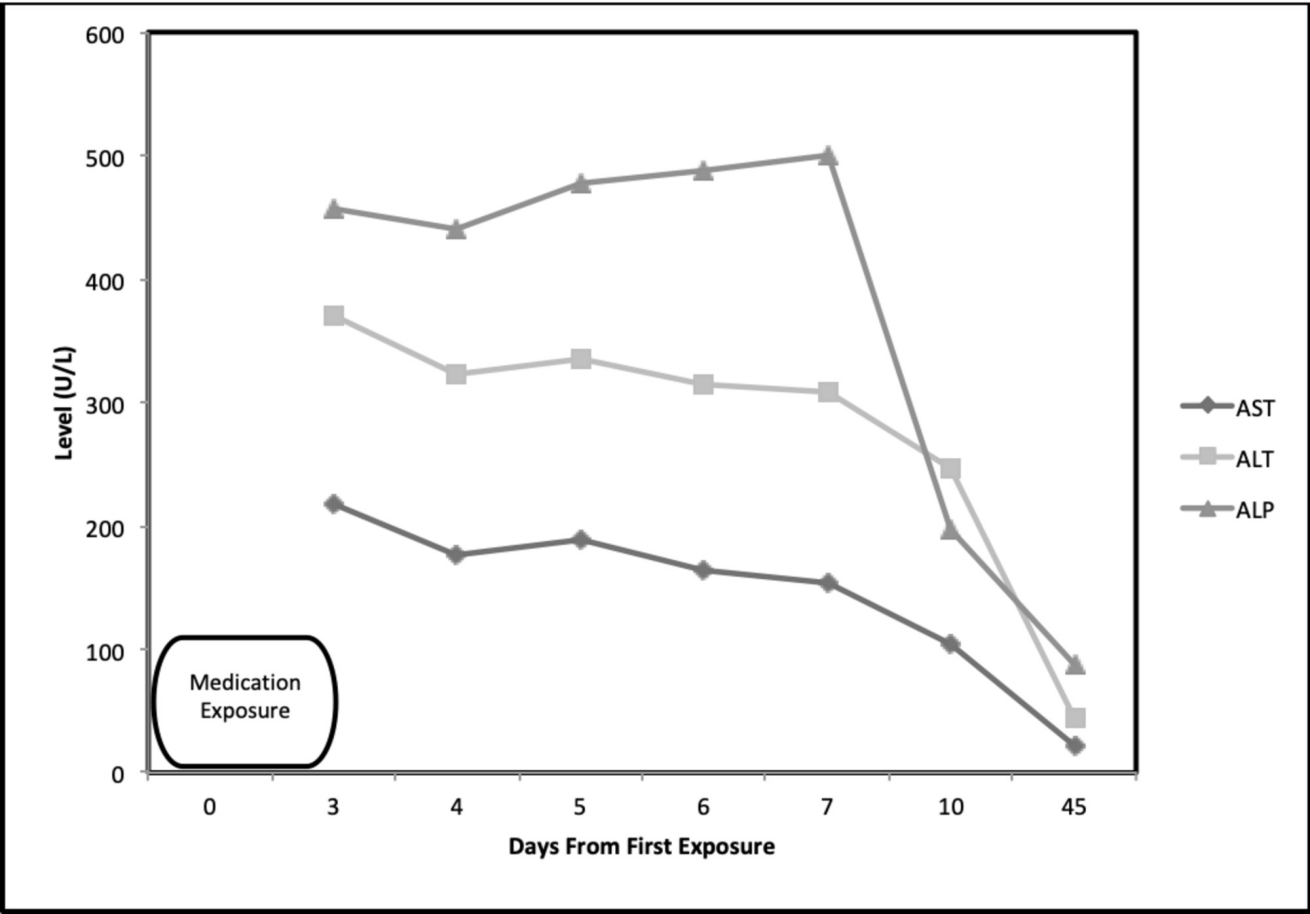
Aspartate aminotransferase (AST), alanine aminotransferase (ALT), and alkaline phosphatase (ALP) levels up to 45 days after exposure to fluticasone-vilanterol inhaler

**Figure 2:**
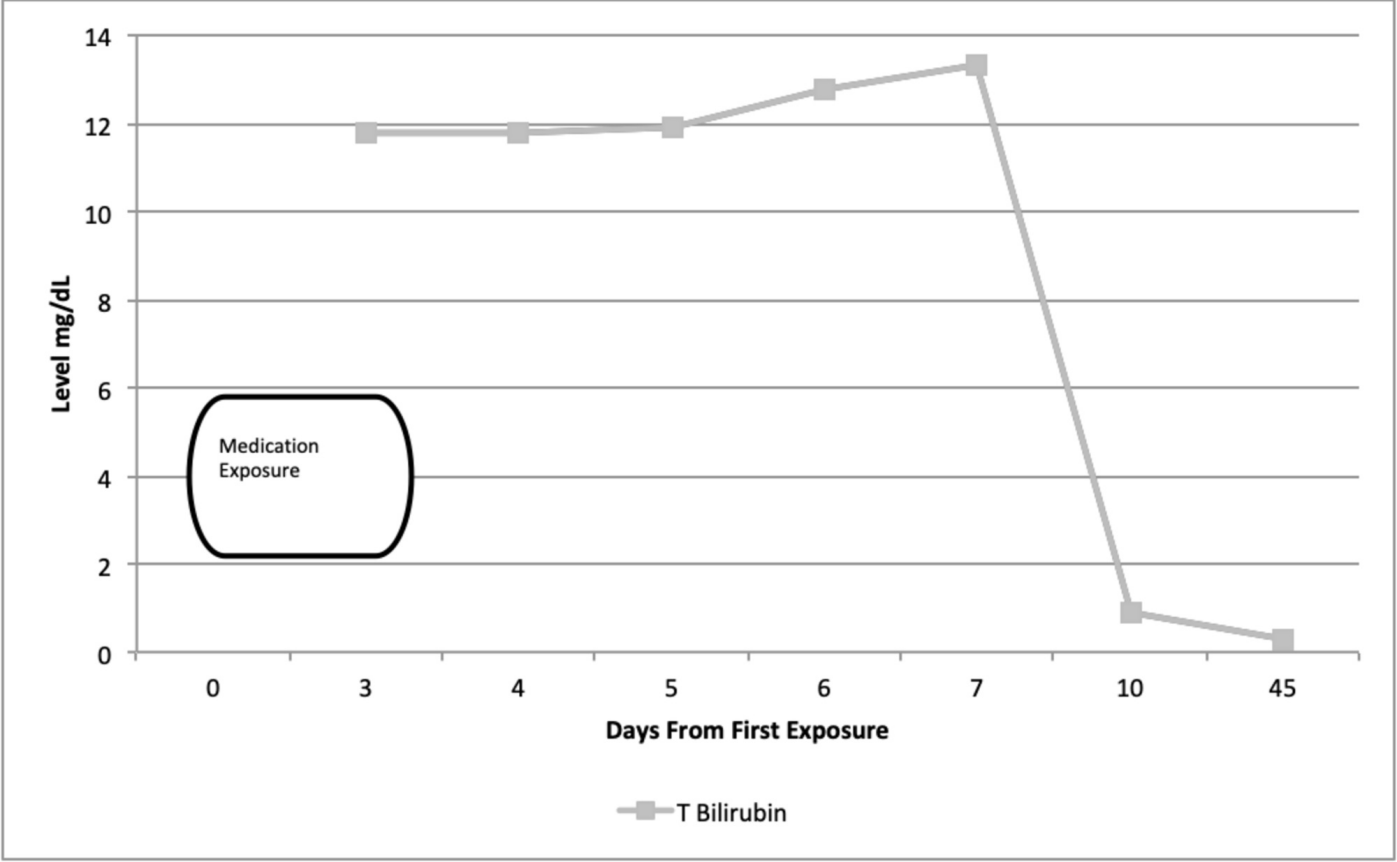
Total bilirubin level up to 45 days after exposure to fluticasone-vilanterol inhaler

**Figure 3:**
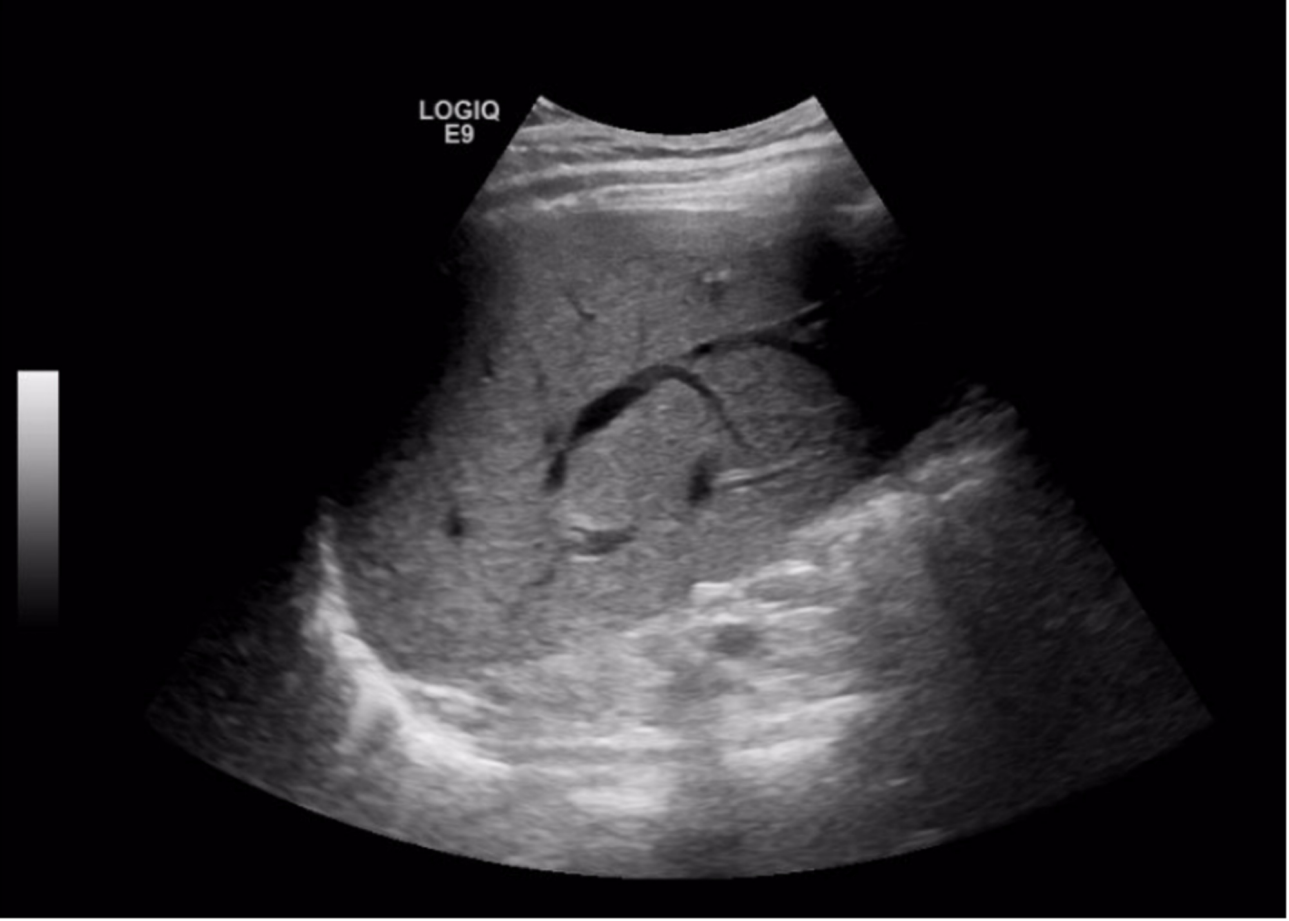
Ultrasound of the liver shows heterogenous liver texture

Prior to starting this new medication, the patient used an albuterol rescue inhaler and glycopyrrolate-formoterol fumarate combination for management of COPD. The latter medication was switched to fluticasone-vilanterol by his outpatient pulmonologist. Three days after starting this new medication, the patient noticed skin rashes, dyspnea on exertion, progressive nausea, vomiting, diarrhea, poor appetite, pruritus, anorexia, and weight loss. His condition deteriorated to the point that he was lethargic and icteric. Given these symptoms, the patient stopped taking this new medication and presented to the emergency department for evaluation. Upon admission, liver chemistries were performed, which showed the following: aspartate aminotransferase (AST) = 206 U/L (normal 12-55 U/L), alanine aminotransferase (ALT) = 371 U/L (normal 2-50 U/L), alkaline phosphatase (ALP) = 456 U/L (normal 30-99 U/L), total bilirubin (T Bilirubin) = 11.9 mg/dL (normal 0.1-1.5 mg/dL), direct bilirubin = 9.5 mg/dL (normal 0.1-1.5 mg/dL), albumin = 3.2 g/dL (normal 3.2-4.9 g/dL), and gamma-glutamyl transferase (GGT) = 800 U/L (normal 9-48 U/L) (Figures 1-2). International normalized ratio (INR) was normal at 0.97 (normal 0.87-1.13). His white blood cell count and hemoglobin were within normal limits. He presented with acute kidney injury with a creatinine of 1.51 mg/dL (elevated from his baseline of 1.0 mg/dL) (normal 0.5-1.4 ml/dL) and blood urea nitrogen of 34 mg/dL (8-22 mg/dL). The patient also presented with non-anion gap metabolic acidosis with a bicarbonate level of 17 mEq/L (normal 22-28 mEq/L) secondary to acute kidney injury. Urinalysis was positive for bilirubinuria. No peripheral eosinophilia was noted. Serum acetaminophen was undetectable on admission. Viral hepatitis serologies were negative for hepatitis B (HBV) sAg, hepatitis C (HCV) Ab, and hepatitis A (HAV) Ab. A urine drug screen was negative for cannabis, opiates, benzodiazepines, and cocaine metabolites. Ultrasound of the abdomen was completed and revealed heterogeneous liver texture, hepatopedal portal vein flow, contracted gallbladder without biliary ductal dilatation, and normal partially visualized pancreas (Figures 3).

**Figure 4:**
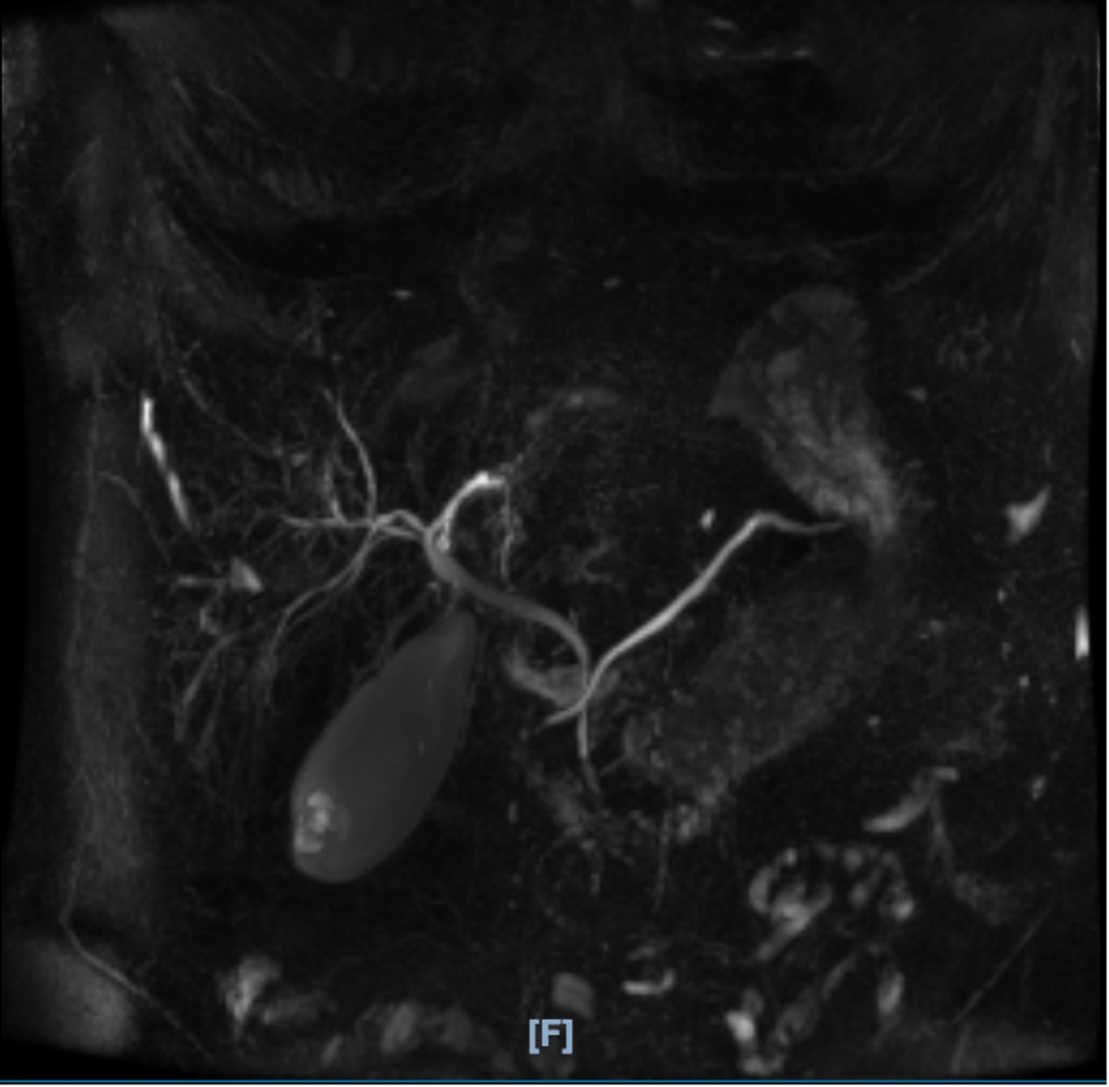
Magnetic resonance cholangiopancreatography (MRCP) MRCP was not suggestive of biliary obstruction or intrahepatic biliary ductal dilatation

**Figure 5:**
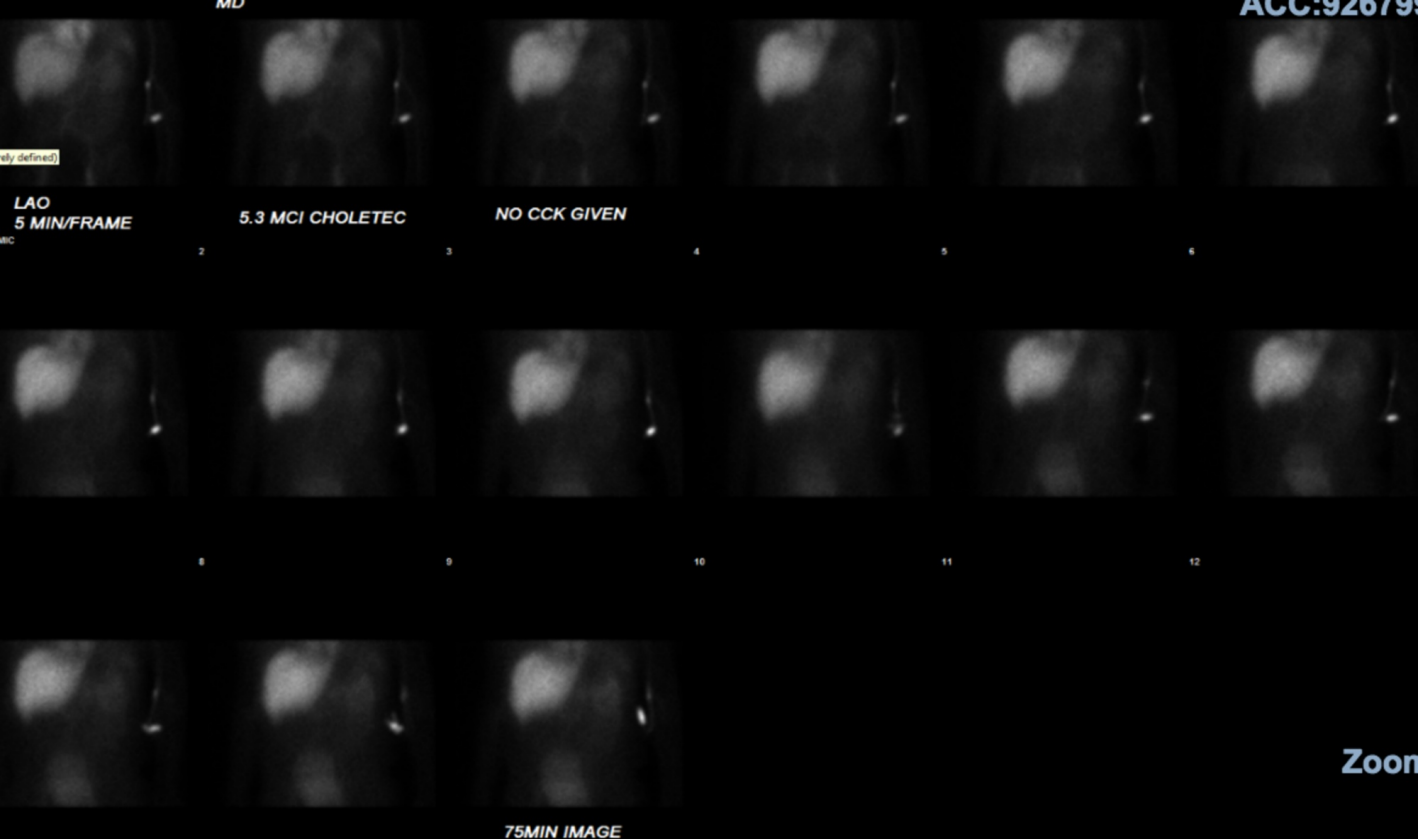
Cholescintigraphy with 99m technetium Hepatobiliary scan shows prompt hepatic uptake, prolonged hepatic activity, lack of hepatic transit, and lack of hepatic excretion—findings suggestive of primary hepatic dysfunction

Due to the significant hepatocellular dysfunction along with cholestatic picture at presentation, magnetic resonance cholangiopancreatography (MRCP) was performed to rule out biliary obstruction. It showed gallbladder sludge without choledocholithiasis, pancreatic divisum, mild hepatomegaly, and small renal cysts (Figure 4). The common bile duct (CBD) measured 7 mm proximally and tapered at the pancreatic head (normal 4-8 mm). There was no intrahepatic biliary ductal dilatation. Chronic liver disease workup including celiac panel (tissue transglutaminase, endomysial antibody, and total IgA), smooth muscle antibody titer, antinuclear antibody titer, and hemochromatosis panel (ferritin and iron levels) was normal. After discussion with the radiology department, a hepatobiliary scan (HIDA) was performed, which showed prompt hepatic uptake, but prolonged hepatic activity and lack of hepatic transit/excretion (Figure 5). The results were consistent with diffuse hepatocellular injury. The biliary products and gallbladder were not seen due to lack of radionucleotide excretion; therefore, cystic or biliary duct obstructions were not excluded. Due to the possibility of choledocholithiasis or sludge not seen on MRCP, endoscopic ultrasound with endoscopic retrograde cholangiopancreatography was considered for biliary decompression. However, the patient refused. Ultrasound-guided liver biopsy was performed through interventional radiology. Liver biopsy showed a predominance of eosinophils in the portal tracts compatible with a drug reaction. The portal tracts were moderately inflamed and showed features of mixed inflammatory infiltrate with lymphocytes, eosinophils, and rare neutrophils. No lobular or interface hepatitis was present. Lobular hepatocytes featured increased deposition of lipofuscin in the cytoplasm but no canalicular bile plugs to indicate cholestasis. No steatosis, ballooning degeneration, or necrosis of the hepatocytes was appreciated. Iron and periodic acid-Schiff staining (PAS) were normal. According to Batts-Ludwig criteria, activity grade was 2 and fibrosis stage was 0.

The patient’s hepatic panel normalized one week after stopping the offending agent. There was a concomitant improvement of his symptoms of pruritus, lethargy, and generalized weakness. The patient was asked to report fluticasone-vilanterol as an allergy. Re-challenge was not performed.

## Discussion

DILI is a rare disorder that is associated with acute liver failure and even death. Due to its low incidence in the general population, this disorder is considered a diagnosis of exclusion. Hepatotoxicity can be classified as an intrinsic (non-idiosyncratic) dose-dependent reaction or an idiosyncratic DILI wherein the reaction is dose-independent and more varied in presentation and course. Observation made by Hyman Zimmerman suggests a 10% mortality when certain criteria are met during presentation, including serum ALT >3 x upper limits of normal (ULN), serum total bilirubin elevated to >2 x ULN (without initial cholestasis findings), and a presentation without a secondary cause for hepatocellular injury [7,8]. While the majority of patients with DILI clinically recover, around 9.2% either die or require liver transplantation [9]. As expected, a high Model For End-Stage Liver Disease (MELD) score (which is calculated using the patient's bilirubin, creatinine, and international normalized ratio [INR]) is independently associated with short term mortality with DILI [10]. The components of the MELD score are as follows: a high score is associated with a poorer prognosis. The overall disease spectrum is broad. The expectation for recovery even among jaundiced individuals is 90% and initial cholestatic injury carries a better prognosis compared to a hepatocellular injury pattern. There is an increased risk of developing DILI with increasing age due to medications such as isoniazid, amoxicillin-clavulanate, and nitrofurantoin. In addition, herbal supplements, including flavoxocid and prohormone supplements, have been associated with the development of DILI [3]. In a prospective study recruiting patients suspected of drug-induced liver injury, 45% of the cases were attributed to antimicrobials and 15% were attributed to central nervous system acting agents among the 100 different medications, herbal supplements, and dietary supplements implicated [11].

The diagnostic approach to drug-induced liver injury is tailored according to the pattern of liver injury at presentation. The “R-value” is defined as alanine aminotransferase/upper limit of normal divided by serum alkaline phosphatase/upper limit of normal. An R-value above 5 indicates hepatocellular damage; R = 5-2 indicates mixed picture, and R<2 is suggestive of cholestatic DILI. Although it has not yet been validated, the R-value is commonly used in clinical practice to identify patterns of acute liver injury [3]. Furthermore, excluding secondary causes of liver dysfunction is important. In patterns suggestive of hepatocellular injury, viral hepatitis, cytomegalovirus, and Epstein Barr virus serologies should be performed. For a predominantly cholestatic picture, abdominal ultrasound, cholangiogram, or endoscopy should be performed based to exclude biliary obstruction. In this case, the R-value was suggestive of mixed DILI. Multiple assays and imaging studies including a hepatitis panel, autoimmune panel, abdominal ultrasound, HIDA scan, and MRCP were performed and were negative for secondary causes. A liver biopsy can be considered in cases in which the timeline of the onset of DILI from the first day of the suspected agent consumption is obscure. Other indications for biopsy include a lack of clinical or laboratory improvement after removal of the offending agent or to rule out autoimmune hepatitis, such as in this case [12]. Extensive testing was negative for secondary causes for hepatocellular injury or biliary obstruction. Although liver biopsy is not mandatory for the evaluation of DILI, severe cases likely have higher biopsy rates due to referral bias. Persistent elevation of elevated transaminases often lowers the threshold for biopsy [3].

The Roussel Uclaf Causality Assessment Method (RUCAM) System is a commonly used method of assigning points for chemical, clinical, serological, and radiological features of liver injury to assess causality in cases suspected of DILI [13,14]. The total score consists of points allotted to each individual suspected drug for causality assessment. A score of 0 to 8 is given correlating with unlikely, possible, probable, and highly probable association with liver injury. In this case, fluticasone-vilanterol was indicated as the probable cause of liver injury. Of note, deliberate re-challenge with fluticasone-vilanterol was not performed due to the concerns for patient safety in acute hepatocellular injury. Although this is an integral part of the RUCAM assessment, it is rarely done. Furthermore, the reliability of the RUCAM is controversial, and thus the diagnosis of DILI is often established based on clinical judgement and expert opinion [15].

Fluticasone is metabolized through CYP3A4 and rates of total body clearance are equivalent to hepatic blood flow [16]. Vilanterol is metabolized primarily in the liver through O-dealkylation and metabolites are excreted through urine [17]. Although this patient did not have underlying liver disease, the manufacturer does not recommend dosage adjustments based on hepatic impairment. This case has been reported to MedWatch. Future DILI events need to be reported and monitored closely.

## Conclusions

Drug-induced liver injury (DILI) due to prescription and non-prescription substances represents a significant cause of liver disease. Diagnosis is often challenging, and establishing a causal relationship to suspected agents is required due to the lack of confirmatory tests. Other common and infrequent causes of liver injury need to be ruled out as DILI is an exclusionary diagnosis. Patient presentation and outcomes are highly variable with DILI although avoidance of the offending agent is vital for recovery.
